# The complete chloroplast genome of *Myxopyrum hainanense* and phylogenic analysis of Oleaceae

**DOI:** 10.1080/23802359.2020.1763864

**Published:** 2020-05-18

**Authors:** Xingfu Zhu, Zhongming Xiong, Kaili Yang, Xihan Li

**Affiliations:** School of Life Sciences, Guizhou Normal University, Guiyang, China

**Keywords:** Chloroplast, *Myxopyrum hainanense*, phylogenetic analysis

## Abstract

We assembled and characterized the complete chloroplast genome sequence of *Myxopyrum hainanense* to investigate its phylogenetic position. The plastome is 156,064 bp in length, which is comprised of a large single-copy (LSC) region of 86,851 bp, a small single-copy (SSC) region of 17,837 bp, and two inverted repeat (IR) regions of 25,688 bp. The overall GC content of the plastome was 37.7. The new sequence comprised total 135 genes, including 87 protein-coding genes, 8 ribosomal RNA genes, and 40 tRNA genes. Phylogenetic analysis showed that *M. hainanense* was close to *Nyctanthes arbor-tristis.*

The Oleaceae is a family of flowering plants in the order Lamiales, which consists of about 700 species in 25 extant genera (Green 2004). Many species are economically important, such as the olive (*Olea europaea*), the ash tree (*Fraxinus excelsior*) and numerous ornamentals or fragrant species (i.e., the genera *Forsythia*, *Ligustrum*, *Jasminum*, *Osmanthus,* and *Syringa*). Here, we report the first complete plastome of *Myxopyrum hainanense,* an endemic shrub in Hainan, China and we constructed a phylogenetic tree including 23 of the 25 extant genera to investigate its position (GenBank accession number: MN908148).

The leaves of *M. hainanense* were sampled from South China Botanical Garden, Guangzhou, China (23.1928°N,113.3706°E) and the voucher specimen was deposited at the Herbarium of Guizhou Normal University (Accession number: Zhu201905005). We extracted total genomic DNA with the Qiagen DNeasy Plant Mini Kit (Qiagen, Carlsbad, CA, USA), and performed the subsequent high-throughput sequencing on an Illumina Hiseq 2500 System. We assembled the plastome using GetOrganelle v1.6.2e (Jin et al. 2018). Annotation was performed using PGA (Qu et al. 2019) and manually corrected.

The plastome is 156,064 bp in length, which is comprised of a large single-copy (LSC) region of 86,851 bp, a small single-copy (SSC) region of 17,837 bp, and two inverted repeat (IR) regions of 25,688 bp. The new sequence comprised total 135 genes, including 87 protein-coding genes, 8 ribosomal RNA genes, and 40 tRNA genes. In these genes, ten protein-coding genes (atpF, ndhA, ndhB, petB, petD, rpl16, rpl2, rps12, rpoC1, rps16) contained one intron and two genes (clpP and ycf3) contained two introns. The overall percentage of GC content was 37.7, and the corresponding value of the LSC, SSC, and IR region were 35.2, 31.5, and 43.2, respectively.

To further investigate the phylogenetic position of *M. hainanense,* a maximum likelihood tree was constructed based on 22 published complete plastomes of Oleaceae and the new plastome. The published chloroplast genome sequences of *Silvianthus bracteatus* (Carlemanniaceae) was defined as outgroup, because the Carlemanniaceae family was a sister group to the Oleaceae (Stevens 2017; Zhu et al. 2020). We using RAxML (Stamatakis 2014) to construct the tree after the sequences were aligned using MAFFT v7.307 (Katoh and Standley 2013). Our results showed that *M. hainanense* was close to *Nyctanthes arbor-tristis* (Figure 1). This published *M. hainanense* chloroplast genome will provide useful information for phylogenetic and evolutionary studies in Oleaceae.

**Figure 1.**
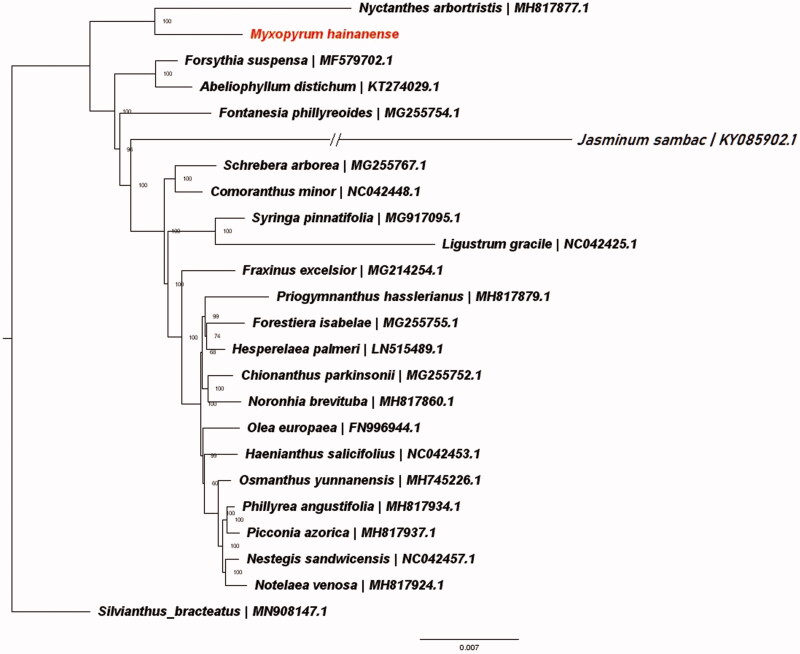
ML phylogenetic tree of the Oleaceae based on the 22 chloroplast genome sequences in GenBank, plus the chloroplast sequence of *Myxopyrum hainanense*. The tree is rooted with the Carlemanniaceae (*Silvianthus bracteatus*). Bootstraps (10000 replicates) are shown at the nodes.

## Data Availability

The data that support the findings of this study are openly available in GenBank (https://www.ncbi.nlm.nih.gov/) with accession number MN908148.
